# Mind–Body Intervention for Post-Surgical Breast Cancer Patients in Southern Italy: A Pilot Feasibility Study of Qigong on Patient-Reported Symptom Burden and Emotional Well-Being

**DOI:** 10.3390/cancers18172758

**Published:** 2026-08-25

**Authors:** Graziella Marino, Eugenia Giglio, Martina Giuseffi, Giovanni Pace, Carlo Calabrese, Marzia Sichetti, Marisabel Mecca

**Affiliations:** 1 Unit of Breast Cancer, Centro di Riferimento Oncologico Della Basilicata (IRCCS-CROB), 85028 Rionero in Vulture, Italy; graziella.marino@crob.it; 2 Laboratory of Preclinical and Translational Research, Centro di Riferimento Oncologico Della Basilicata (IRCCS-CROB), 85028 Rionero in Vulture, Italy; eugenia.giglio@crob.it (E.G.); marisabel.mecca@crob.it (M.M.); 3 Transfusion Unit, “San Carlo” Hospital, 85100 Potenza, Italy; martina.giuseffi@ospedalesancarlo.it; 4 Associazione AGATA OdV ETS, 75015 Marconia di Pisticci, Italy; giovannipace67@gmail.com; 5 Scientific Directorate, Centro di Riferimento Oncologico Della Basilicata (IRCCS-CROB), 85028 Rionero in Vulture, Italy; carlo.calabrese@crob.it

**Keywords:** breast cancer, supportive care, Qigong, mind–body intervention, integrative oncology, symptom burden, quality of life

## Abstract

Background: Post-surgical breast cancer (BC) survivors frequently experience clusters of persistent post-treatment physical and psychological symptoms, which can negatively affect their quality of life (QoL). Mind–body interventions such as Qigong may offer potential benefit, but evidence in oncology remains limited. Methods: We conducted a single-arm pilot feasibility study at the AMICO clinic (IRCCS-CROB, Southern Italy) to evaluate the feasibility, acceptability, safety, and exploratory pre–post symptoms associated with an 8-week Qigong programme in post-surgical BC patients. Fourteen women (aged 42–73 years; stage I–III) who reported symptom burden and emotional sensitivity attended weekly one-hour classes and were encouraged to practise at home. Feasibility outcomes included adherence, completion of post-intervention assessment, adverse events, and participant acceptability. Symptom severity was assessed at baseline and post-intervention using a 0–5 study-specific symptom questionnaire score. For the secondary descriptive prevalence analysis, scores ≥ 3 were classified as indicating moderate-to-severe symptom burden. Results: All 14 participants completed the post-intervention assessment, overall intervention adherence was 92%, and no adverse events were reported. In the secondary descriptive analysis, the prevalence of moderate-to-severe pain and mood changes decreased from 64.3% to 35.7%, fatigue from 57.1% to 28.6%, anxiety from 85.7% to 42.9%, and sleep disturbances from 35.7% to 14.3%. Hot flushes decreased from 64.3% to 42.9%. Despite the heterogeneity of individual symptom trajectories, most participants reported meaningful improvements in overall well-being. Conclusions: The 8-week programme was feasible, well tolerated, and acceptable in this small real-world cohort. Symptom burden decreased during the intervention period; however, because of the uncontrolled study design and small sample size, these changes cannot be attributed specifically to Qigong. Larger controlled studies are required to estimate treatment effects and identify potential moderators of response.

## Simple Summary

Breast cancer survivors often continue to experience pain, fatigue, sleep problems, hot flashes, anxiety and emotional distress after surgery, even when their cancer treatment has been successful. These symptoms can reduce quality of life and are not always adequately managed by standard medical care. This pilot study explored whether Qigong, a gentle practice that combines slow movements, breathing exercises, and relaxation, could help improve physical and emotional well-being in women recovering from breast cancer surgery. Fourteen women participated in an eight-week Qigong program delivered within a public integrative oncology clinic. Most participants reported improvements in pain, fatigue, anxiety, mood, sleep, and overall well-being, and the program was safe and well accepted. Although this was a small pilot study, the findings suggest that Qigong may be a useful supportive therapy alongside conventional cancer care and provide a strong basis for larger clinical studies to confirm its benefits.

## 1. Introduction

Breast cancer (BC) is the most prevalent malignancy among women, and survival has improved markedly due to advances in early diagnosis and multimodal treatment [1,2]. However, many women experience persistent physical and psychological sequelae after surgery and adjuvant therapies (e.g., pain, fatigue, sleep disturbance, vasomotor symptoms, anxiety, and low mood), which can substantially impair quality of life (QoL) during the early survivorship phase [2,3,4,5]. To overcome these adverse effects, non-pharmacological supportive interventions have gained more attention in routine BC patients’ survivorship care, owing to their safety and minimal side effects.

In particular, as QoL is now acknowledged as a crucial outcome measure in both clinical trials and survivorship studies, the inclusion of integrative approaches in routine cancer care has grown in importance. Integrative oncology is a patient-centred, evidence-informed approach to cancer care that integrates conventional oncology treatments with selected complementary interventions to address the physical, psychological, and social dimensions of living with cancer. In clinical practice, its role is not to “replace” standard therapies but to support patients alongside surgery, chemotherapy, radiotherapy, endocrine or targeted treatments by improving symptom control and day-to-day functioning (e.g., fatigue, pain, sleep disturbance, anxiety, mood symptoms). Interventions are chosen based on the best available evidence, safety, patient preferences, and clinical context. By offering structured, coordinated supportive care across the cancer treatment continuum and survivorship, integrative oncology aims to improve QoL and the overall patient experience while maintaining alignment with guideline-concordant cancer treatment [6,7,8,9].

Estimates suggest that approximately 33% to 47% of cancer patients utilise complementary, alternative, or integrative medicine. More than 80% of BC survivors report having used integrative therapies during their cancer treatment journey [10,11,12].

The National Centre for Complementary and Integrative Health (NCCIH) classifies integrative therapies into two main categories [13]. The first one includes natural products, such as herbs, vitamins, minerals, and probiotics (often sold as dietary supplements). The second category encompasses mind–body practices, which include yoga, chiropractic and osteopathic manipulation, meditation, massage therapy, acupuncture, relaxation techniques, Tai chi, and Qigong.

As a matter of fact, several studies demonstrated the effectiveness of exercise, nutritional therapy, and psychological interventions in reducing both psychological (e.g., anxiety, mood disturbances, melancholy) and somatic (e.g., pain, fatigue) symptoms among BC patients [14,15,16]. In particular, exercise is increasingly recognised as an important treatment in the recovery and rehabilitation of cancer survivors [17] offering benefits such as improved physical performance, body composition, and better overall QoL [18,19,20]. Starting from these assumptions, there has been a notable shift toward holistic approach, particularly mind–body therapies, that address both physical and emotional dimensions of survivorship.

These innovative forms of mind–body therapies stem from the growing interest in meditative movement, which integrates physical postures or gentle movements with focused breathing to cultivate a meditative state [6]. Meditative movement often includes a “flowing motion” [21], with low-to-moderate levels of exertion, and includes practices such as Yoga, Tai Chi, and Qigong [22].

Among mind–body practices, Qigong—a traditional Chinese therapy with a history spanning over 5000 years—has gained increasing attention in supportive cancer care [23]. The term “Qigong” is composed of two Chinese characters: “qi” (pronounced “chi”) denotes the vital energy that flows through the human body; while “gong” (pronounced “gung”) alludes to the systematic exercise aimed at directing and enhancing this vital energy to specific areas of the body [24].

The rationale behind selecting Qigong over other mind–body approaches is because it is considered a primary mind–body intervention due to its “meditative movement” practice that combines gentle physical activity with breath regulation and attentional training, aligning well with common post-surgical survivorship needs (e.g., co-occurring somatic symptoms, emotional distress, and reduced functional confidence). Compared with approaches that are predominantly cognitive (e.g., mindfulness-only) or more physically demanding (e.g., vigorous yoga-based programs), Qigong can be delivered at low intensity, requires no equipment, and can be adapted to variable postoperative limitations (e.g., shoulder mobility restrictions), supporting feasibility and safety in early survivorship.

Qigong involves slow, deliberate movements that are synchronised with controlled breathing and a variety of cognitive skills. The goal is to strengthen, relax, and harmonise both body and mind for better health and personal development [25]. Its low-intensity and ease of practice make Qigong particularly suitable for BC patients, who may experience fatigue or reduced physical capacity and are often less likely to enjoy or benefit from intense, complex activities [26,27,28].

Despite strong evidence supporting the use of physical activity in managing and alleviating post-treatment symptoms [29], Qigong, a practice involving light physical activity and meditative movements, remains under-represented in current clinical practice guidelines. Moreover, research specifically examining Qigong interventions in oncology (particularly among BC survivors) remains limited [30,31]. Importantly, while previous studies have examined the effects of Qigong on cancer-related fatigue, mood disturbances, and quality of life, fewer have investigated the broader burden of persistent patient-reported physical and psychological symptoms experienced during the early post-surgical phase of breast cancer survivorship. These symptoms, which include pain, fatigue, vasomotor complaints, sleep disturbance, gastrointestinal symptoms and emotional distress, often coexist and may arise from multiple interacting mechanisms, including surgery, systemic therapies, menopause and psychological adaptation to cancer. Moreover, evidence remains scarce regarding the implementation of Qigong within publicly funded integrative oncology services and its real-world feasibility, limiting the ability to determine how Qigong may improve clinical outcomes and cancer-related endpoints. As a result, there is a lack of evidence for determining how Qigong improves clinical outcomes and cancer-related endpoints. In light of these considerations, there is a clear need to develop and implement new projects to promote mind–body practices in several oncology care settings.

The primary aim of this single-arm pilot study was to evaluate the feasibility, acceptability, and safety of implementing an 8-week Qigong programme within a hospital-based integrative oncology service for post-surgical BC survivors. A secondary exploratory aim was to characterise within-participant changes in physical and emotional symptom burden over the intervention period.

## 2. Materials and Methods

### 2.1. Study Design

The study was designed as an exploratory, single-arm pilot trial to evaluate the feasibility and acceptability of the 8-week Qigong programme. The primary outcomes were assessed through recruitment, adherence, completion rate, adverse events, and participant satisfaction. The study focused on BC patients who were still experiencing significant overall physical and psychological distress during and after standard oncological therapies. Secondary outcomes included changes in overall physical and psychological symptom burden, assessed using the study-specific symptom questionnaire. Baseline in-person assessments collected oncological history and clinical variables, including haematological analysis, age, body mass index (BMI), body fat percentage, tumour histology and grade, hormonal/HER2 status, type of surgery, menopausal status, ongoing systemic therapies, and lifestyle factors (smoking and alcohol use), as summarised in Table 1. The post-intervention assessment (T2) was administered immediately after completion of the 8-week Qigong programme. The study was approved by the Ethics Committee for Basilicata (CEUR 47/2023, no. 20240003417).

### 2.2. Participants

For this pilot study BC patients were recruited from the Breast Unit of the Basilicata Oncology Reference Centre (IRCCS-CROB) in Rionero in Vulture, southern Italy. The enrolled patients were women diagnosed with stage I to III BC who had undergone surgical treatment and were experiencing persistent patient-reported physical and psychological symptoms within three months post-surgery. Eligible patients were screened based on self-reported symptom burden and sensitive emotional state, and no minimum pain severity was required for participation.

**Figure 1:**
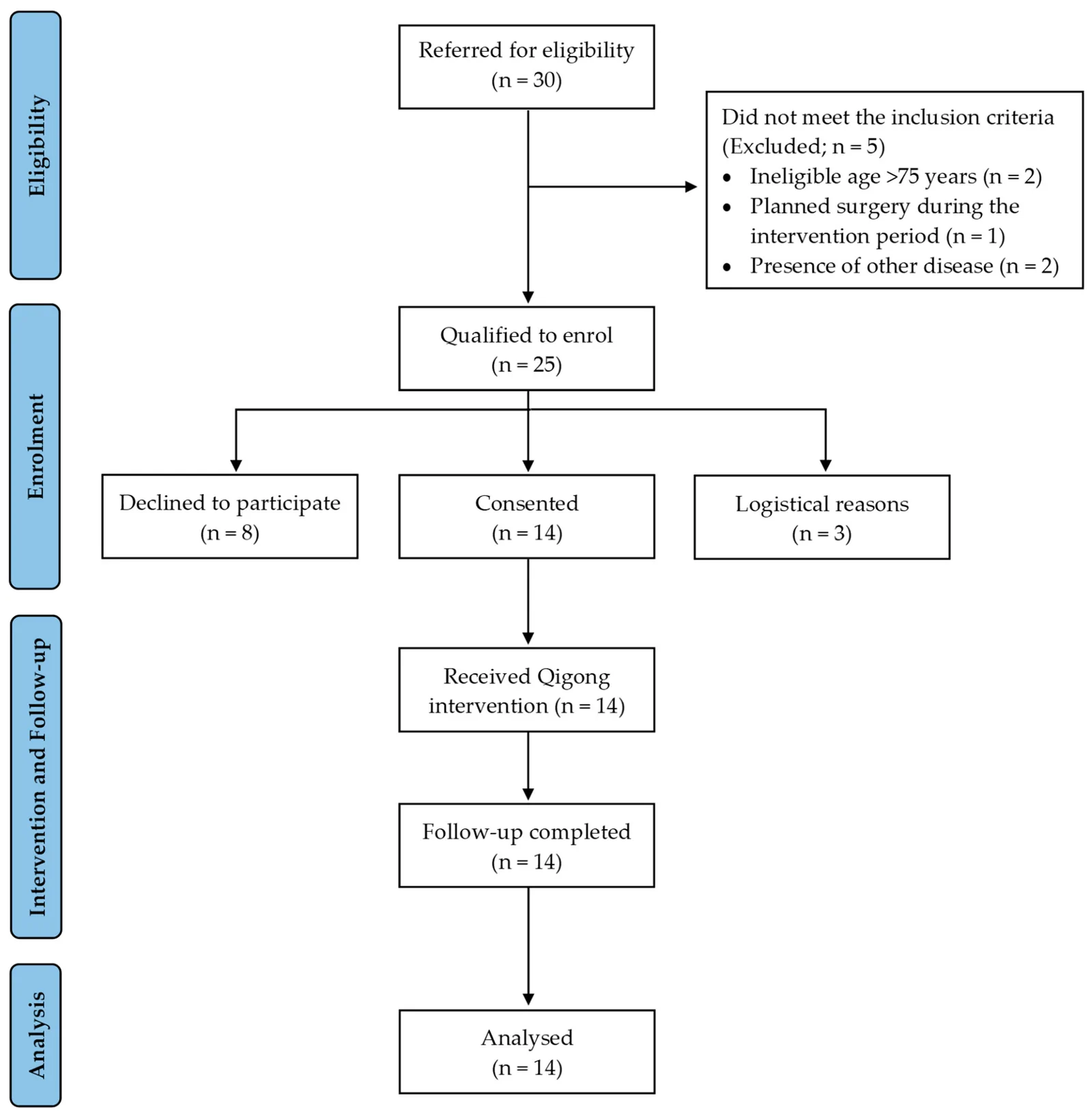
Flow diagram of participant recruitment, enrolment, intervention, and analysis. Thirty women with post-surgical breast cancer were screened for eligibility. After exclusion of women who did not meet the eligibility criteria or declined participation, 14 participants were enrolled in the study. All participants completed the 8-week supervised Qigong program and the post-intervention assessment, with no losses to follow-up.

Our target sample size was 30 women, who were screened for eligibility between June 2024 and August 2024. Of these, 25 met the eligibility criteria; overall, 14 consented and enrolled in the Qigong programme in September 2024, 8 declined participation, and 3 could not enrol for logistical reasons (Figure 1).

Each participant received an information sheet, two copies of the informed consent form, and the adapted symptom assessment questionnaire.

Listed below are the broadest inclusion and exclusion criteria established by the multidisciplinary team.

*Inclusion Criteria:*

(1) Aged between 25–75 years old;
(2) Diagnosed with Stage I–III BC;
(3) 2 weeks to 3 months post-surgical intervention;
(4) Able to independently read and answer questionnaires in English or Italian language;
(5) Experiencing symptom burden and fatigue, along with heightened emotional sensitivity, as assessed by adapted symptom assessment questionnaire;
(6) Estimated life expectancy of at least 3 months;
(7) Able to provide written informed consent;
(8) Willing and able to comply with all the study procedures and attend scheduled sessions.

*Exclusion Criteria*

(1) Presence of cardiopulmonary disease; nerve, muscle, or joint disorders; metastatic cancer; or other active malignancies;
(2) History of chronic medical conditions that might affect upper extremity function (e.g., stroke, Parkinson’s disease, multiple sclerosis), or any serious cognitive impairment and defects in language that significantly impair communication;
(3) History of post-operative complications involving heart, cerebral vessel, or other serious complications;
(4) Diagnosed mental illness or neurodegenerative disease (e.g., dementia), leading to reduced cognitive capacity that could affect the ability to understand trial procedures or the ability to provide informed consent;
(5) Inability to ambulate independently;
(6) Planned surgery during the intervention period;
(7) Currently pregnant;
(8) Any other medical conditions which would preclude study intervention or make study participation unsafe.

### 2.3. Procedures

The “AMICO” clinic (acronym for Ambulatorio di Medicina Integrata e Condotta in Oncologia), located within the Breast Unit of the Basilicata Oncology Reference Centre (IRCCS-CROB) in Rionero in Vulture, southern Italy, represents a pioneering initiative within the context of a public healthcare system, offering post-surgery BC patients unrestricted cost-free access to integrative therapies [32]. In this context, a breast surgeon specialised in integrative oncology therapies and lifestyle medicine, supported by a multidisciplinary team of researchers and a certified Qigong instructor, performs a comprehensive assessment of each patient with BC. This assessment includes an in-depth review of the patient’s oncological history, detailed clinical data on the oncological condition, current health status and overall QoL. Following this initial assessment, the team recommends low-intensity physical activity and Qigong sessions, together with lifestyle recommendations aimed at supporting quality of life and supporting recovery.

After being enrolled, patients attended Qigong classes held at the IRCCS-CROB facilities, which are easily accessible to the community, and were encouraged to attend classes at least once a week. Classes were offered after work hours and on weekdays to accommodate participants’ work or family schedules.

To facilitate participation and maintain a small-group format, the 14 participants were allocated to one of three parallel Qigong classes comprising five, five, and four participants, respectively. Each class met once weekly for a 1-h supervised session, over eight consecutive weeks. Therefore, three supervised classes were scheduled each week, corresponding to 24 class sessions at the programme level, while each participant was scheduled to attend 8 supervised sessions in total. Classes were offered on weekdays and, where possible, outside standard working hours to accommodate participants’ work and family commitments.

Attendance at each supervised session was recorded by the Qigong instructor. Participant adherence was defined as the proportion of the eight scheduled supervised sessions attended by each participant. Overall supervised-session adherence was calculated from the total number of participant attendances relative to the total number of scheduled participant attendances across the cohort. Home-based Qigong practice was recorded separately and was not included in the supervised-session adherence calculation.

To evaluate the impact of the intervention on patient-reported symptom burden and emotional state, participants were asked to fill out adapted symptom assessment questionnaires to collect baseline (T1) and 8-week post-intervention follow-up (T2) data. No additional, structured, supportive intervention was introduced within the AMICO clinic during the 8-week study period. Nevertheless, concurrent oncological treatments, spontaneous post-surgical recovery, individual lifestyle changes, expectation effects and other non-specific influences could not be controlled and may have contributed to the observed symptom trajectories.

Moreover, brief in-person exit interviews were conducted upon completion of the program to gather qualitative feedback on participants’ experiences and perceptions of the Qigong intervention.

### 2.4. Development of the Symptom Assessment Questionnaire

A structured symptom assessment questionnaire was developed for the purposes of this pilot feasibility study, using the Edmonton Symptom Assessment System (ESAS) and the 12-Item Short Form Health Survey (SF-12) as conceptual frameworks. Selected domains relevant to post-surgical breast cancer survivorship were incorporated into a single study-specific questionnaire, together with additional clinically relevant symptoms frequently observed in routine integrative oncology practice. These additional items included hot flushes, bowel habit changes, tachycardia, dysphagia/nausea, sleep disturbance, and melancholy.

Upon completion of the 8-week programme, participants were invited to take part in brief semi-structured exit interviews of 12 questions addressing various aspects of physical and mental health (e.g., physical activity, pain, fatigue, and emotional well-being) to assess acceptability and perceived impact. For the purposes of this study, symptom burden was defined as the presence of persistent patient-reported physical or psychological symptoms occurring during the early post-surgical period, irrespective of their underlying cause. Because several symptoms may reflect surgery, ongoing systemic therapy, menopause, or other recovery-related factors, no attempt was made to classify symptoms as medically unexplained. Interviews were conducted in person in a private setting by a trained researcher not involved in delivering the classes, using an interview guide covering (i) overall acceptability and satisfaction, (ii) perceived physical and emotional changes, (iii) feasibility/barriers to attendance and home practice, (iv) perceived safety/adverse experiences, and (v) intention to continue practice.

Each symptom was rated using a study-specific 6-point ordinal symptom-burden scale ranging from 0 (absence of the symptom) to 5 (persistent and severely disabling symptom). This simplified scoring system was adopted to facilitate symptom monitoring within routine clinical practice and to ensure consistent assessment across all symptom domains. An overall symptom burden score was calculated at the participant level by summing the 11 symptom items reported in Table 2 and Table 3, with higher values indicating greater symptom burden. For secondary descriptive analyses only, each symptom was dichotomised using the prespecified threshold of <3 versus ≥3, with scores ≥ 3 classified as moderate-to-severe symptom burden.

Because the questionnaire was specifically developed for this pilot feasibility study, no formal psychometric validation (e.g., reliability or construct validity testing) was performed. Consequently, findings derived from this instrument should therefore be interpreted as exploratory and hypothesis-generating.

### 2.5. Qigong Intervention

The Qigong intervention followed a standardised multiple mind–body exercise protocol that was taught by an instructor who was certified to deliver “Qigong Easy”. This technique was developed by the Institute of Integral Qigong and Tai-Chi, which is based on a small number of Qigong movements as an integrated practice that are taught in a series of repeated and easy-to-learn movements [33].

During the first session, the Qigong instructor conducted a standardised functional and safety screening to guide individual tailoring and to accommodate individual functional limitations. The screening assessed upper-limb/shoulder pain and range of motion, posture and balance, breathing patterns, potential contraindications (e.g., cardiopulmonary limitations), and perceived exertion. Findings were documented using a structured checklist and used to adjust movement range, pace, and postures for each participant.

Following this assessment, the instructor incorporated elements of Qigong Easy together with the eight Strands of the Brocade (also known as Ba Duan Jin), a traditional sequence of eight gentle, rhythmic upper-body movements typically delivered at low-to-moderate intensity depending on pace and range of motion, which does not elicit a noticeable increase in heart rate and breathing while still allowing conversation (talk test), consistent with standard definitions. Qigong movements emphasise postural alignment, diaphragmatic breathing, and mindful awareness. These exercises integrate core therapeutic elements, including flowing movement, focused attention, imagery, and present-moment awareness, with the goal of inducing a calm, meditative state and improving both physical function and emotional regulation.

In addition to diaphragmatic breathing, the practice included exercises for controlling the centre of gravity which were also combined with “Dan Tian”. The term “Dan Tian” is used in Chinese medicine to refer to the “body’s energy centre”, located in the lower abdomen approximately an inch or two below the navel [34].

Participants’ functional demands, such as restrictions in stamina and fatigue, were taken into consideration when designing changes to the program’s content and delivery. These included modifying meditation postures, including lying, sitting, or utilising furniture or a wall for support.

All supervised sessions were delivered by the same certified Qigong instructor according to a predefined standardized protocol. No protocol deviations occurred, and all planned intervention components were delivered throughout the study.

Attendance at supervised Qigong sessions was recorded by the instructor at each class. Participants were also encouraged to perform home-based Qigong practice using the instructional materials provided.

To assess the acceptability of the Qigong intervention, brief in-person exit interviews were conducted at the end of the 8-week program to gather participant perceptions and opinions about the classes.

### 2.6. Home Practice

Participants, provided with individualised home practice recommendations during each class, were encouraged to practise at home daily for approximately 30–45 min with a minimum target of 2–3 h/week. Every participant received guided video meditations to facilitate engagement at home.

Adherence to home practice was monitored through structured self-report journals, in which participants documented frequency, duration, and perceived physical and emotional responses. This self-monitoring was intended to enhance self-awareness and provide additional insight into participants’ responses to the intervention. Individual self-report journals were not sufficiently standardised or complete to derive a quantitative adherence measure; therefore, adherence calculations were based exclusively on supervised-session attendance.

### 2.7. Statistical Analysis

All data were screened prior to analysis using range and consistency checks. All analyses were performed on available data without imputation and were considered exploratory. Feasibility outcomes, including recruitment, adherence, completion of post-intervention assessments, adverse events, and participant acceptability, were summarised descriptively. Clinical outcome analyses were restricted to participants with paired baseline (T1) and post-intervention (T2) observations. Distributional assumptions were evaluated using visual inspection (histograms and boxplots) and, where appropriate, normality tests.

The original 0–5 symptom scores were retained for the primary, exploratory, longitudinal analyses. Individual symptom scores and the participant-level overall symptom burden score were summarised using median and interquartile range (IQR). Within-participant change in overall symptom burden was evaluated using a two-sided Wilcoxon signed-rank test. The unit of analysis was the participant (n = 14 paired observations).

For secondary sensitivity analyses, symptom scores were dichotomised as <3 versus ≥3. For each symptom, baseline and post-intervention prevalence, the numbers of participants changing from ≥3 to <3 and from <3 to ≥3, and the absolute change in percentage points were reported. Paired binary comparisons were evaluated using the two-sided exact McNemar test. Given the sparse number of discordant pairs, these analyses were interpreted descriptively, rather than as confirmatory hypothesis tests. Within-participant change in the overall symptom burden score was evaluated using a two-sided Wilcoxon signed-rank test, with the participant set as the unit of analysis (N = 14 paired observations). For the overall symptom burden score, the Wilcoxon test statistic (W), exact two-sided *p*-value, paired sample size, and standardised effect-size estimate (r = |Z|/√N, where Z is the standardised Wilcoxon statistic and N is the number of paired observations) were reported. The magnitude of the paired change was additionally estimated using the Hodges–Lehmann estimator of location shift (T2 − T1), with its 95% confidence interval. Negative estimates indicate lower symptom burden at T2.

For individual symptom scores, baseline and post-intervention values were summarised as median and interquartile range (IQR). Hodges–Lehmann paired location-shift estimates with 95% confidence intervals and Wilcoxon-derived standardised effect-size estimates (r = |Z|/√N) were reported for descriptive purposes. Symptom-specific Wilcoxon *p*-values were not reported or interpreted, because these analyses were exploratory, involved multiple outcomes, and were not intended to provide confirmatory inference.

Between-group comparisons across independent subgroups (e.g., tumour grade II vs. III; presence vs. absence of a given symptom) were conducted using Mann–Whitney U tests (two-sided). Associations among age, physical activity, and dichotomised symptom indicators were explored through correlation matrices computed on coded variables (0/1 for dichotomised symptoms; continuous age), reported as Pearson correlation coefficients (equivalent to φ coefficients for binary pairs) and visualised using heatmaps. The correlation matrix was used exclusively for descriptive pattern visualisation and hypothesis generation. Given the small sample size and the large number of pairwise comparisons relative to the number of participants, no correlation-specific hypothesis testing was performed, and no *p*-values were used to classify individual associations as statistically significant. Correlation coefficients were therefore interpreted cautiously and were not considered evidence of reproducible symptom clusters or mechanistic relationships. Radar charts were used to visualise pre–post changes in symptom prevalence at the cohort level.

All analyses and graphs were generated using GraphPad Prism (version 10, GraphPad Software, San Diego, CA, USA). No adjustment for multiple comparisons was applied, because symptom-specific and subgroup analyses were exploratory. Accordingly, individual *p*-values for secondary/exploratory comparisons were interpreted descriptively and in conjunction with effect magnitude and clinical relevance.

### 2.8. Qualitative Data Analysis

The semi-structured exit interviews were transcribed and anonymised prior to analysis. Participants’ responses were entered into a structured Microsoft Excel database organised according to the broad domains defined in the interview guide a priori: (i) overall acceptability and satisfaction, (ii) perceived physical and emotional changes, (iii) feasibility and barriers to attendance and home practice, (iv) perceived safety and adverse experiences, and (v) intention to continue Qigong practice. These predefined domains provided the deductive framework for the analysis.

A combined deductive–inductive content analysis approach was then applied. Within each predefined domain, participants’ responses were iteratively reviewed to identify recurrent concepts, experiences and patterns that emerged directly from the data.

These inductively generated codes were compared across participants and progressively grouped into broader categories and themes according to their conceptual similarity and frequency of occurrence.

Qualitative analysis was conducted by two researchers, M.S. and M.M., who both had two years’ experience of working with a physician specialising in integrative therapies within the integrative oncology research team. Both researchers reviewed the transcribed responses, contributing to the coding process, categorisation and the development of the final themes. Coding decisions and theme definitions were discussed jointly, and any differences in interpretation were resolved through discussion and consensus. As the analysis was based on consensus coding rather than a formal assessment of independent coder agreement, no inter-rater reliability coefficient was calculated.

The resulting themes were then reviewed against the original responses to ensure they accurately reflected the participants’ accounts.

## 3. Results

### 3.1. Demographic and Clinical Characteristics of the Study Cohort

The study cohort comprised 14 women with breast cancer (BC) who had undergone surgical treatment and were receiving different adjuvant treatments. Of the 30 women screened for eligibility, 25 met the inclusion criteria and 14 ultimately enrolled, corresponding to 46.7% of the screened population and 56.0% of eligible participants. Table 1 summarises the main demographic, anthropometric, tumour-related, treatment, and lifestyle characteristics of the study population.

**Figure 2:**
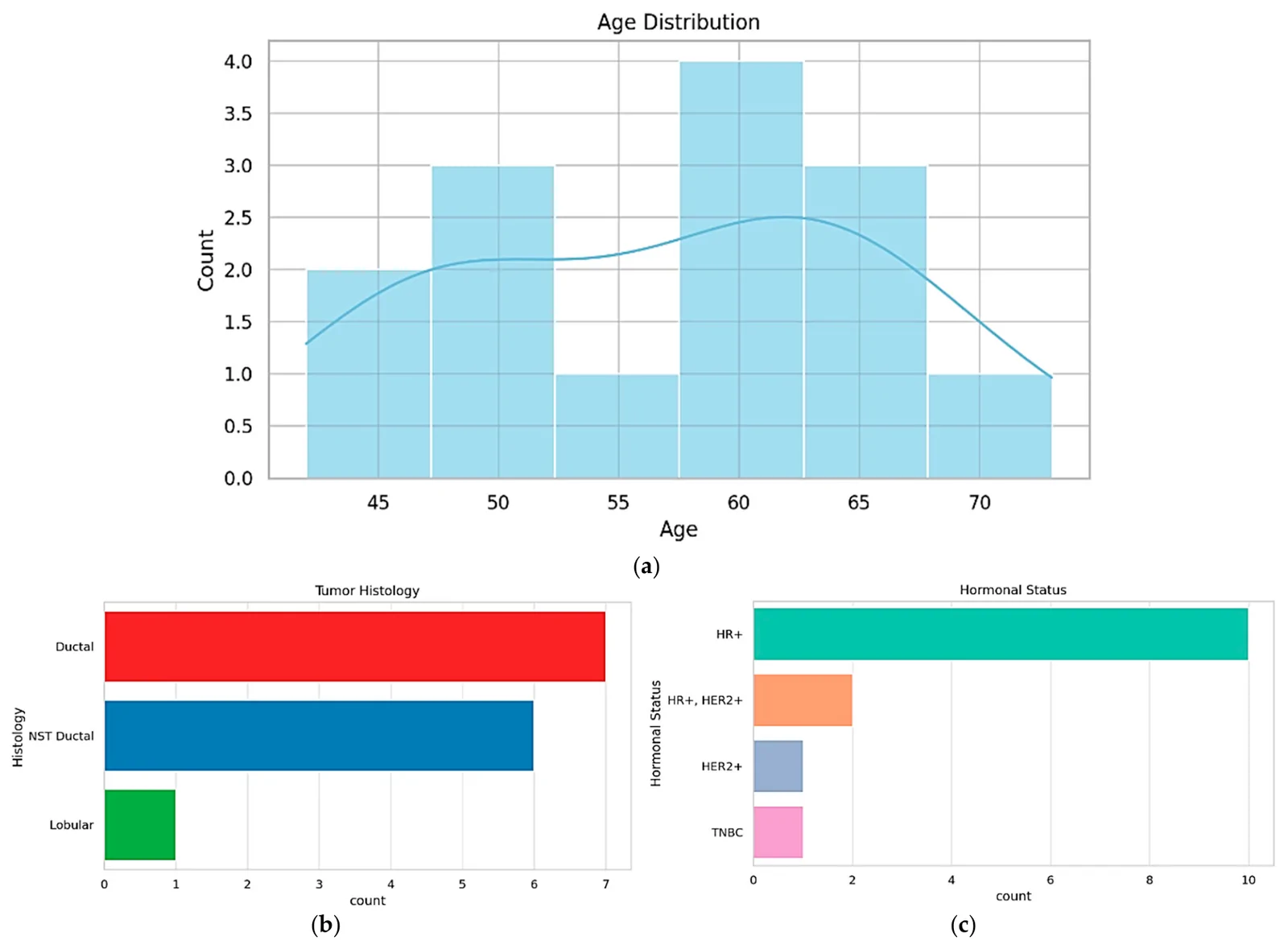
Graphical representation of key cohort characteristic: age distribution (**a**), tumour histology (**b**), and hormonal status (**c**) of BC Patients.

Participants ranged in age from 42 to 73 years, with a mean age of 57.4 years, and most participants were between 45 and 65 years of age (Figure 2a). Twelve of the fourteen participants (85.7%) were postmenopausal. BMI categories were defined according to NIH/WHO cut-offs (underweight < 18.5; normal 18.5–24.9; overweight 25.0–29.9; obesity ≥ 30 kg/m^2^). BMI values (Table 1) ranged from the normal-weight to the obese range, with overweight being the most frequently represented BMI category (BMI ≥ 25 kg/m^2^). Body fat percentage (BFP) ranged from 15.4% to 43.1%, indicating substantial inter-individual variability in body composition within the cohort (Table 1).

Regarding tumour characteristics (Table 1), Grade II tumours were the most common, accounting for 9 of the 14 participants (64.3%), whereas the remaining 5 participants (35.7%) presented Grade III tumours. No Grade I tumours were represented. Ductal and No Special Type (NST) ductal carcinoma together accounted for 92.9% of cases, whereas one participant had a lobular tumour (Figure 2b). Hormone receptor-positive (HR^+^) tumour was present in 12 participants (85.7%), Human Epidermal growth factor Receptor 2 positive (HER2^+^) tumour was present in 3 patients (21.4%), and one participant (7.1%) had Triple-Negative Breast Cancer (TNBC) (Figure 2c).

Among the surgical interventions, breast-conserving surgery was more frequently performed than mastectomy, with mastectomy accounting for 35.7% of surgical procedures. Ongoing systemic treatments reflected tumour subtype and receptor status. Letrozole, an aromatase inhibitor, was the most frequently prescribed endocrine therapy, whereas patients with HER2^+^ tumours received trastuzumab-based regimens, with pertuzumab and docetaxel. Pembrolizumab, an immune checkpoint inhibitor, was administered exclusively to the single patient with TNBC.

Regarding lifestyle characteristics, none of the participants reported current smoking, whereas three (21.4%) reported alcohol consumption during meals.

**Figure 3:**
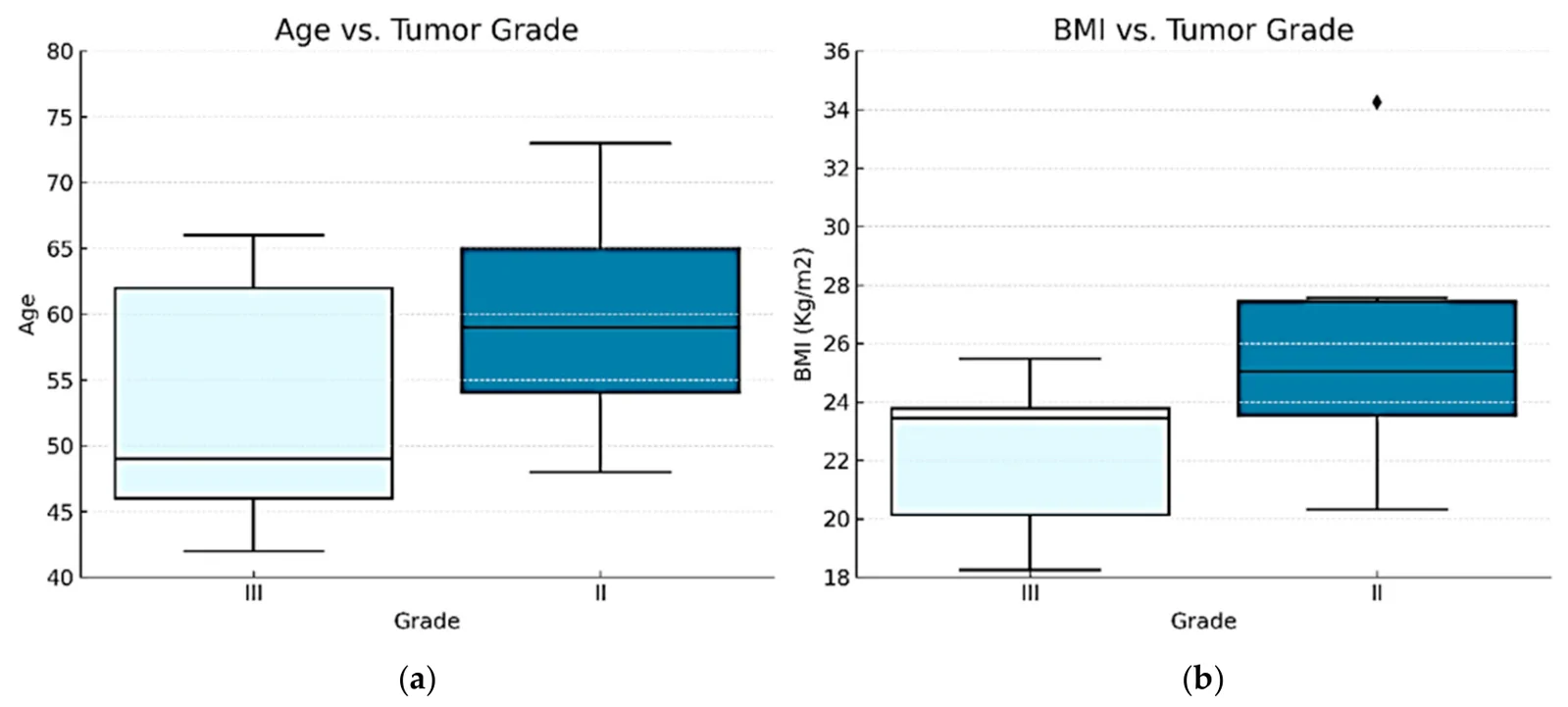
Distribution of age (**a**) and BMI (**b**) according to tumour grade in the study cohort. Individual observations are shown for participants with Grade II and Grade III tumours. Horizontal lines indicate median values. Between-group comparisons were performed using the Mann–Whitney U test and were considered exploratory.

To further characterise the cohort, age and BMI distributions were explored according to tumour grade (Figure 3). Participants with Grade II tumours had a higher median age than those with Grade III tumours (approximately 59 versus 49 years, respectively); however, the distributions showed substantial overlap and the between-group comparison was not statistically significant (Mann–Whitney U test, *p* = 0.349; Figure 3a).

Similarly, median BMI was slightly higher among participants with Grade II tumours than among those with Grade III tumours (approximately 25 versus 23 kg/m^2^), but this difference was also not statistically significant (Mann–Whitney U test, *p* = 0.112; Figure 3b).

Given the small number of participants within each tumour-grade category, these comparisons were considered exploratory and are presented solely to descriptively characterise the cohort.

### 3.2. Baseline Symptom Profile and Exploratory Symptom Co-Occurrence

Table 2 shows the individual baseline symptom severity scores, which were assessed using a 0–5 scale. At T1, participants exhibited a heterogeneous but generally substantial symptom burden, with an overall median symptom burden score of 28.5 (IQR 19.0–34.25; range 11–40) across the 11 assessed symptoms. The highest median severity scores were observed for anxiety (median 4.0, IQR 3.0–4.75) and mood changes (median 3.5, IQR 2.0–4.0). Hot flushes (median 3.0, IQR 2.0–4.0), pain (median 3.0, IQR 1.25–4.75) and fatigue (median 3.0, IQR 1.25–4.0) also contributed substantially to the baseline symptom profile (Table 4).

**Figure 4:**
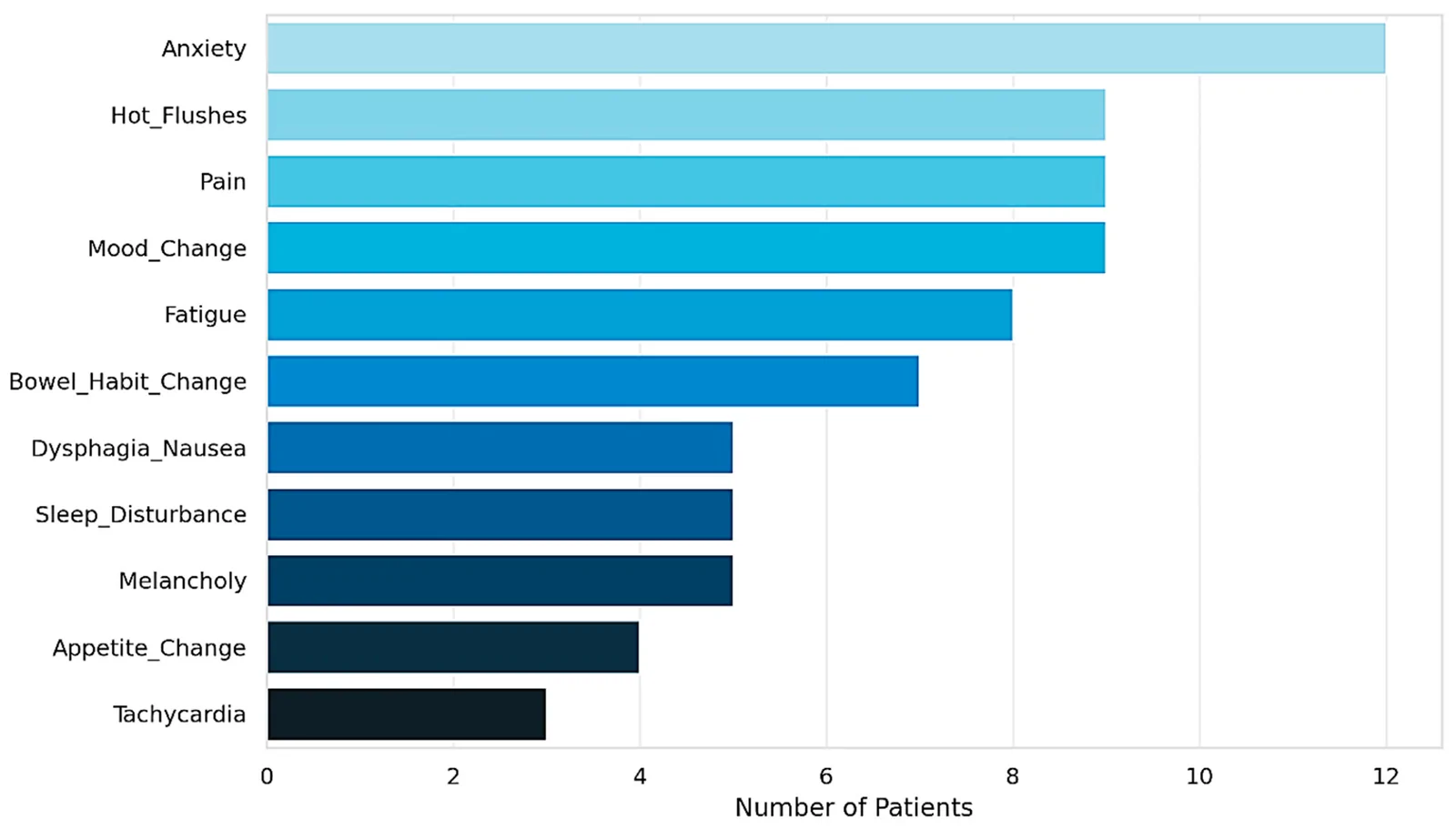
Prevalence of moderate-to-severe symptoms at baseline (T1) among post-surgical breast cancer participants. Bars represent the percentage of participants with a symptom-severity score ≥ 3 on the 0-5 scale. Symptoms are ordered according to decreasing prevalence.

For descriptive purposes, symptom scores were additionally classified according to the prespecified threshold of ≥3, indicating moderate-to-severe symptom burden. Anxiety was the most frequently reported moderate-to-severe symptom, affecting 12 of 14 participants (85.7%). Hot flushes, pain, and mood changes were each reported by 9 participants (64.3%), followed by fatigue in 8 (57.1%) and changes in bowel habits in 7 (50.0%). Dysphagia/nausea, sleep disturbances, and melancholy were each present at moderate-to-severe levels in 5 participants (35.7%), whereas appetite changes and tachycardia were less frequent, affecting 4 (28.6%) and 3 (21.4%) participants, respectively (Figure 4).

With respect to habitual physical activity, 11 of the 14 participants (78.6%) reported no regular physical activity at baseline, whereas three participants reported regular walking. Physical activity was self-reported and was not assessed using a validated physical activity instrument; therefore, these data were considered descriptive only.

**Figure 5:**
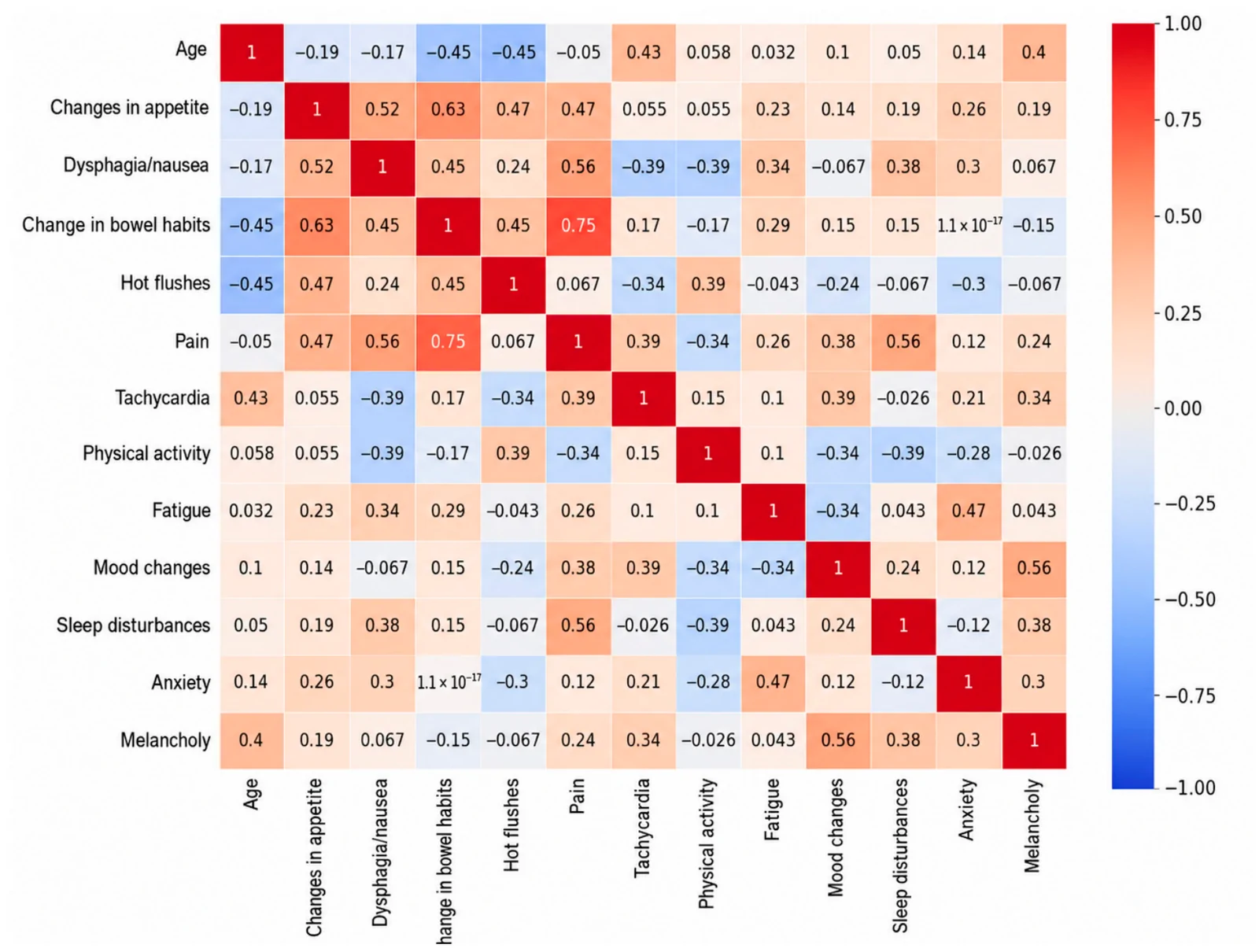
Exploratory heatmap of baseline symptom co-occurrence. Phi coefficients (φ) were calculated for pairwise associations between physical activity and dichotomised symptom variables (score <3 vs. ≥3), whereas Pearson/point-biserial coefficients (r) were used for associations involving age. The heatmap is presented for descriptive and hypothesis-generating purposes.

To further explore symptom co-occurrence within the cohort, pairwise associations among dichotomised baseline symptom variables were visualised using a correlation heatmap (Figure 5). Among symptom pairs, the largest positive φ coefficient was observed between pain and changes in bowel habits (φ = 0.75), followed by appetite changes and bowel-habit changes (φ = 0.63). Positive coefficients were also observed between mood changes and melancholy (φ = 0.56), pain and dysphagia/nausea (φ = 0.56), and pain and sleep disturbances (φ = 0.56). These patterns indicate that some symptoms tended to co-occur within this small cohort, although they should not be interpreted as evidence of formally established symptom clusters.

Hot flushes showed an inverse association with age (r = −0.65), while smaller inverse coefficients were observed between hot flushes and tachycardia (φ = −0.34), anxiety (φ = −0.30), and mood changes (φ = −0.24). In a post hoc exploratory comparison, participants with moderate-to-severe hot flushes were younger than those without moderate-to-severe hot flushes (median age: 49 versus 65 years, respectively; Mann–Whitney U = 6, *p* = 0.032).

Physical activity also showed inverse coefficients with sleep disturbances (φ = −0.39), mood changes (φ = −0.34), and pain (φ = −0.34). Given the small sample size (n = 14) and the number of pairwise comparisons, these coefficients were considered descriptive and hypothesis-generating only. Accordingly, they were not interpreted as evidence of stable symptom clusters, independent associations, or causal relationships.

### 3.3. Feasibility Outcome and Exploratory Pre–Post Symptom Changes

At the end of the 8-week Qigong programme, we evaluated feasibility outcomes and exploratory within-participant changes in patient-reported symptoms burden and emotional states between baseline (T1) and post-intervention assessment (T2) (Table 4). All 14 participants enrolled in the study completed the 8-week intervention and the post-intervention (T2) assessment. Adherence was calculated as the percentage of scheduled supervised Qigong sessions attended by participants. Overall attendance was an average of 22 out of the 24 scheduled sessions, corresponding to an average adherence rate of approximately 92%. Participants were also encouraged to practice Qigong independently at home using the provided instructional materials; however, home practice was self-recorded in participant journals and was not sufficiently standardized or complete to derive a quantitative adherence measure. Therefore, adherence calculations were based exclusively on supervised-session attendance. Throughout the study, no intervention-related adverse events or protocol deviations were observed.

Analysis of the original 0–5 symptom scores showed a consistent reduction in overall symptom burden over the study period. The participant-level overall symptom burden score, calculated as the sum of the 11 symptom items (possible range: 0–55), decreased from a median of 28.5 (IQR 19.0–34.25) at T1 to 17.0 (IQR 11.75–26.25) at T2. The Hodges–Lehmann paired shift was −8.0 points (95% CI −10.5 to −6.5). The Wilcoxon signed-rank test yielded W = 0.0 (n = 14 paired observations; exact two-sided *p* = 0.00012), with a large within-participant effect-size estimate (r = 0.88). All 14 participants showed a lower overall symptom burden at T2 than at T1, with individual reductions ranging from 4 to 16 points. Because the study did not include a concurrent control group, these findings represent within-participant longitudinal changes and should not be interpreted as a causal effect of Qigong.

Changes were also observed across several individual symptom domains (Table 4). Anxiety showed the largest descriptive reduction in severity, decreasing from a median of 4.0 (IQR 3.0–4.75) at T1 to 2.0 (IQR 1.0–3.0) at T2, with a Hodges–Lehmann paired shift of −1.5 points (95% CI −2.0 to −1.0). Mood changes decreased from a median of 3.5 (IQR 2.0–4.0) to 2.0 (IQR 1.0–3.0), corresponding to a paired shift of −1.0 points (95% CI −1.5 to −0.5). Pain and fatigue both decreased from median values of 3.0 at baseline to 2.0 after the intervention, whereas hot flushes decreased from a median of 3.0 to 2.0. Other physical and emotional symptoms showed smaller or more variable changes.

As a secondary sensitivity analysis, symptom scores were dichotomised using the prespecified threshold of ≥3. The largest absolute reduction in the prevalence of moderate-to-severe symptoms was observed for anxiety, which decreased from 85.7% at T1 to 42.9% at T2 (−42.9 percentage points). Pain, fatigue, and mood changes each decreased by 28.6 percentage points. Hot flushes and sleep disturbances decreased by 21.4 percentage points, while smaller reductions were observed for melancholy and bowel-habit changes (−14.3 percentage points each), as well as for appetite changes and dysphagia/nausea (−7.1 percentage points each). The prevalence of tachycardia was unchanged (21.4% at both assessments).

Exact McNemar tests were performed as exploratory paired analyses of these dichotomised outcomes (Table 4). Although the largest imbalance in discordant pairs was observed for anxiety (exact *p* = 0.031), symptom-specific *p*-values were not interpreted as confirmatory evidence because of the small sample size, sparse discordant pairs, and multiple symptom comparisons. Accordingly, the continuous 0–5 scores and the participant-level overall symptom burden were considered more informative for characterising longitudinal change than the dichotomised symptom classifications.

Individual symptom trajectories were heterogeneous, despite the consistent decrease in overall symptom burden. Given the limited sample size, no formal age-stratified, physical-activity, responder/non-responder, or moderator analyses were performed. In particular, the study design does not allow conclusions regarding additive or interactive effects between Qigong and concurrent physical activity.

### 3.4. Visualization of Pre–Post Changes in Symptom Profile

To complement the analysis of the original 0–5 symptom-severity scores, the radar chart provides a descriptive, visual representation of changes in the prevalence of moderate-to-severe symptoms between baseline (T1) and the post-intervention assessment (T2). For this purpose, individual symptom scores were dichotomised according to the prespecified threshold of <3 versus ≥3, and the proportion of participants with scores ≥ 3 was calculated at each time point.

**Figure 6:**
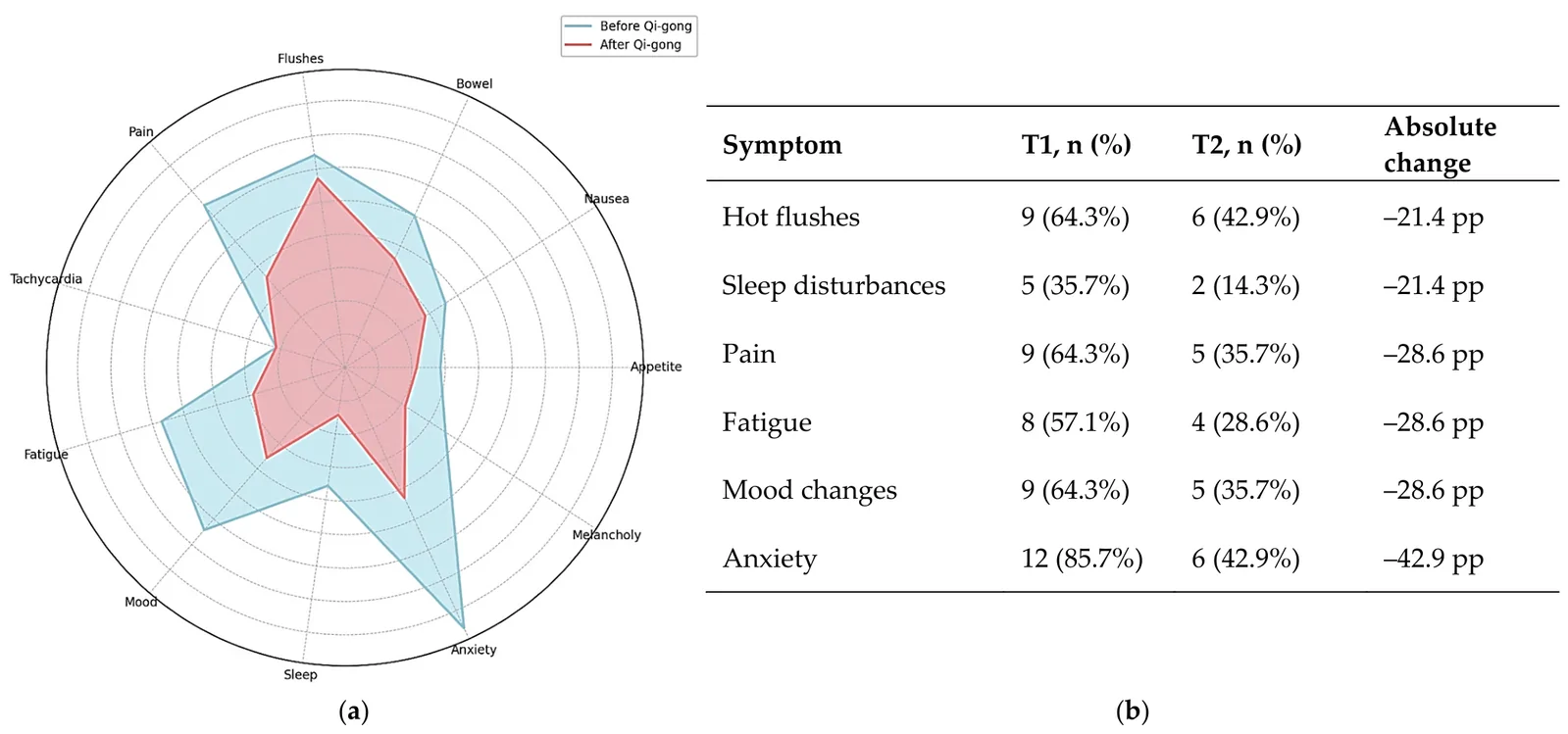
Descriptive changes in the prevalence of moderate-to-severe physical and emotional symptoms between baseline (T1) and the end of the 8-week intervention (T2). (**a**) Radar chart showing the proportion of participants with symptom severity scores ≥ 3 at each assessment. (**b**) Absolute changes in the prevalence of the six symptom domains, showing an absolute decrease of at least 20 percentage points (pp) differences between T1 and T2.

As shown in Figure 6a, the overall symptom profile contracted at T2, although the magnitude of change varied across symptom domains. The largest absolute decrease was observed for anxiety, which declined from 12 of 14 participants (85.7%) at T1 to 6 of 14 (42.9%) at T2, corresponding to a reduction of 42.9 percentage points. Pain decreased from 64.3% to 35.7%, fatigue from 57.1% to 28.6%, and mood changes from 64.3% to 35.7%, each corresponding to an absolute decrease of 28.6 percentage points. Hot flushes decreased from 64.3% to 42.9%, while sleep disturbances decreased from 35.7% to 14.3%, corresponding to reductions of 21.4 percentage points in both cases (Figure 6b).

Smaller absolute decreases were observed for melancholy (35.7% to 21.4%; −14.3 percentage points), changes in bowel habits (50.0% to 35.7%; −14.3 percentage points), appetite changes (28.6% to 21.4%; −7.1 percentage points), and dysphagia/nausea (35.7% to 28.6%; −7.1 percentage points). The prevalence of tachycardia remained unchanged at 21.4%.

The radar chart is intended to illustrate the multidimensional pattern of symptom change, rather than to provide an independent inferential analysis. The corresponding participant-level analysis of the original 0–5 scores showed a decrease in overall symptom burden, from a median of 28.5 (IQR 19.0–34.25) at T1 to 17.0 (IQR 11.75–26.25) at T2, as reported in Section 3.3. Thus, the descriptive contraction of the radar profile is consistent with the overall, within-participant reduction in symptom burden observed over the study period. Given the single-arm design, however, these pre–post changes cannot be attributed specifically to the Qigong intervention.

### 3.5. Qualitative Findings and Participant Acceptability

Qualitative analysis of the post-intervention interviews indicated favourable acceptability and satisfaction with the Qigong programme. All participants completed the exit interview at T2 and reported a positive overall perception of the intervention. Four recurrent themes were identified: (1) emotional regulation and psychological well-being; (2) bodily awareness and perceived physical well-being; (3) social connectedness and shared experience; and (4) engagement in self-care and willingness to continue practice.

Emotional regulation and psychological well-being. Participants frequently described the sessions as promoting calmness and relaxation, with several reporting a greater perceived ability to manage emotional distress, tension, or anxiety. Increased awareness of breathing was also described as a useful component of the practice. These accounts indicate perceived psychological benefits but should not be interpreted as objective evidence of treatment efficacy.

Bodily awareness and perceived physical well-being. Participants reported increased attention to bodily sensations, breathing, posture, and movement, and some described subjective improvements in physical discomfort, fatigue, or sleep. These perceptions complemented the quantitative symptom trajectories observed during the study, but do not independently establish treatment effects.

Social connectedness and shared experience. The group-based format was consistently described as a valuable component of the intervention. Participants highlighted mutual support, shared experiences, encouragement and a sense of belonging, suggesting that the social context contributed to the acceptability of the programme.

Engagement in self-care and continuation of practice. Participants generally considered Qigong compatible with their daily routines and described it as a potentially useful component of their personal self-care. All participants stated that they would recommend the programme to other breast cancer survivors, and most expressed an intention to continue practising Qigong after the study’s completion. Home-based practice was generally perceived as feasible, although some participants reported occasional difficulties in maintaining regular practice because of work or family commitments. Participants also identified practical facilitators and barriers affecting attendance and home practice.

Overall, the interviews supported favourable acceptability and satisfaction, while also indicating variability in participants’ experiences and engagement. Because the interviews were conducted after the intervention, and participants were aware of the study aims, the findings may have been influenced by expectations and social-desirability effects. Accordingly, these qualitative findings are best interpreted as evidence of perceived acceptability and participant experience, rather than as confirmation of clinical efficacy.

## 4. Discussion

This single-arm pilot study evaluated the feasibility, acceptability, and safety of delivering an 8-week Qigong programme within a hospital-based integrative oncology service for women in the early post-surgical phase of breast cancer care. The principal findings support the practical feasibility of the programme, as reflected by complete retention, high supervised-session adherence, the absence of reported intervention-related adverse events, and favourable participant acceptability. Exploratory longitudinal analyses also demonstrated a reduction in overall patient-reported symptom burden over the 8-week study period. While these clinical changes are informative for future research, given the absence of a concurrent control group, they should be interpreted as within-participant observations rather than as evidence of a Qigong-specific treatment effect.

Recruitment was feasible within the hospital-based setting, although participant flow also identified potential challenges for a future definitive trial. Of the 30 women screened, 25 met the eligibility criteria and 14 enrolled in the programme. Eight women met the criteria but declined to participate, and three were unable to enrol due to logistical issues. In contrast, the retention rate following enrolment was successful, with all 14 participants completing the intervention and post-intervention assessment. Alongside the high rate of adherence to supervised sessions (>90%), these findings indicate that the primary feasibility challenge for a larger-scale trial may pertain more to recruitment and initial uptake than to retention once participants have enrolled in the programme. Future studies may therefore benefit from broader referral pathways, longer recruitment windows, or multicentre enrolment. Recruitment, attendance, and adherence have also been identified as relevant feasibility challenges in previous Qigong pilot studies involving breast cancer survivors [35].

From an implementation perspective, the intervention was delivered within an existing public integrative oncology service, which was a further strength [32]. Participants were divided into small, supervised groups, sessions were offered on weekdays and scheduled to accommodate work and family commitments, and movements could be adapted according to individual physical limitations. These organisational features may have contributed to adherence and acceptability, although their relative contribution cannot be separated within the present design. Qigong’s low equipment requirements and adaptable physical demands may nevertheless represent practical advantages when designing supportive interventions for women recovering from breast cancer surgery.

Beyond the feasibility considerations, participants entered the study with a substantial and heterogeneous symptom burden. Anxiety showed the highest baseline severity and prevalence, while pain, mood changes, hot flushes, fatigue, sleep disturbances, and gastrointestinal complaints were also commonly reported. This pattern is consistent with the multidimensional nature of early breast cancer survivorship, during which symptoms may arise simultaneously from surgical recovery, systemic or endocrine treatment, psychological adaptation, menopausal changes, and alterations in daily functioning. The present findings therefore reinforce the clinical importance of evaluating symptom burden across multiple physical and emotional domains rather than considering individual symptoms in isolation [5,36].

The exploratory baseline heatmap further illustrated this co-occurrence. Larger positive coefficients were observed between pain and bowel-habit changes, appetite and bowel-habit changes, mood changes and melancholy, pain and dysphagia/nausea, and pain and sleep disturbances. These findings should not be interpreted as evidence of reproducible symptom clusters because correlation estimates based on only 14 participants are highly sensitive to individual observations. Nevertheless, some descriptive relationships are clinically plausible. In particular, the co-occurrence of pain and sleep disturbance is consistent with the well-described bidirectional relationship between pain and disrupted sleep, whereby persistent discomfort may impair restorative sleep and poor sleep may in turn increase pain sensitivity and fatigue [37]. The exploratory inverse correlation between self-reported physical activity and sleep disturbance is broadly compatible with previous evidence indicating that physical activity may contribute to sleep-related outcomes in breast cancer survivors [38].

The most notable exploratory clinical finding was the reduction in overall symptom burden over the intervention period. The participant-level score decreased from a median of 28.5 at T1 to 17.0 at T2, with a Hodges–Lehmann paired shift of −8.0 pp, and all participants showed a lower total symptom burden at the post-intervention assessment. The consistency of this direction of change is noteworthy for a feasibility study and provides useful preliminary information for the selection of outcome measures and estimation of variability in a future trial. However, the study-specific questionnaire has not undergone formal psychometric validation, and the observed score change cannot currently be translated into a validated minimally important clinical difference. The direction of these exploratory changes is broadly consistent with previous studies of Qigong and related meditative–movement interventions in BC survivorship [30,31,33,39].

At the level of individual symptom domains, anxiety showed the largest descriptive change, decreasing from a median score of 4.0 to 2.0 and from 85.7% to 42.9% when the prespecified moderate-to-severe threshold was applied. Reductions were also observed for pain, fatigue, mood changes, hot flushes, and sleep disturbances. The radar plot provides a complementary visual representation of this multidimensional change, but the dichotomised prevalence estimates should be interpreted as secondary descriptive analyses because categorisation at the ≥3 threshold inevitably reduces information regarding symptom severity.

The observed longitudinal changes are broadly consistent with previous research examining Qigong and related meditative–movement interventions in breast cancer survivorship. Osypiuk et al. reported preliminary findings supporting Qigong as a biopsychosocial intervention for persistent, post-surgical pain in breast cancer survivors [39]. More recently, Chang et al. reported improvements in quality of life and interoceptive awareness following Qigong with breathing meditation in women with breast cancer [31]. An umbrella review and meta-analysis has also suggested potential benefits of Qigong and Tai Chi for quality of life across cancer populations [33]. These studies provide a rationale for continued investigation, but also highlight considerable heterogeneity in intervention type, comparator conditions, participant populations, and outcome measures. Importantly, controlled studies also illustrate why the reductions observed in the present single-arm cohort cannot be assumed to represent Qigong-specific effects. In a recent randomised study by Larkey et al. [30], longitudinal changes in several patient-reported domains were observed across active and comparator conditions. More broadly, the recent Mindfulness and Tai Chi for Cancer Health (MATCH) study demonstrated the clinical relevance of Tai Chi/Qigong and mindfulness-based approaches for addressing psychosocial outcomes among distressed cancer survivors [40]. Although the intervention, study population, and controlled design differ substantially from the present pilot, these findings support continued evaluation of meditative–movement approaches within cancer survivorship care. Such findings emphasise the need for an appropriate comparator, capable of separating intervention-specific effects from attention, expectancy, gentle physical activity, social interaction, and repeated assessment.

Qigong itself is a multicomponent intervention combining low-intensity movement, controlled breathing, attentional regulation, meditative practice, and, in the present programme, a group-based social context. The previous literature has proposed emotional self-regulation, body awareness, attentional control, and stress reduction as possible pathways through which meditative movement may influence patient-reported well-being [31,41]. These mechanisms provide a plausible conceptual framework for the concurrent changes observed across emotional and physical domains. Although physical activity was not systematically quantified to permit formal analysis in the present study, it represents a potentially relevant background factor in cancer survivorship, given its established role in supportive oncology care [42].

The qualitative findings provide additional context for understanding programme acceptability. Four recurring themes emerged from the combined deductive–inductive content analysis: emotional regulation and psychological well-being, bodily awareness and perceived physical well-being, social connectedness and shared experience, and engagement in self-care and willingness to continue practice. These themes complement the quantitative feasibility findings by indicating which aspects of the programme participants perceived as useful or engaging. The reported value of the group setting suggests that social connectedness may itself represent an important component of the intervention experience, rather than merely a background characteristic.

Furthermore, the participants’ descriptions of heightened awareness of breathing, posture, movement, and bodily sensations are consistent with the interoceptive, and self-regulatory dimensions reported in previous Qigong research [31]. However, these qualitative observations represent subjective experiences and should not be used to confirm the quantitative symptom changes. Expectations, willingness to participate in a mind–body programme, and the supportive group environment may all have influenced participants’ accounts. The primary contribution of this study is to reinforce the evidence supporting the acceptability of the intervention, and to identify intervention components that should be investigated prospectively in a larger controlled trial. A future controlled study should also define progression criteria a priori for key feasibility outcomes, including recruitment, retention, adherence, safety, acceptability, and data completeness, consistent with methodological recommendations for pilot and feasibility research [43,44].

The high acceptability observed in this study further supports the feasibility of implementing Qigong within an integrative oncology setting. Participants consistently described the intervention as enjoyable, easy to follow, and compatible with their daily routines, while emphasising the psychological value of the group sessions, enhanced body awareness, and their willingness to continue practising beyond the study period. These findings suggest that Qigong may represent a sustainable, supportive intervention for breast cancer survivorship, supporting translation into integrative oncology pathways, although confirmation in larger, controlled studies is warranted.

Overall, the study provides a useful foundation for the next stage of investigation. A definitive trial should retain feasibility outcomes, such as recruitment, retention, adherence, safety, and acceptability, while incorporating intervention delivery, context, participant experience, and implementation processes, consistent with contemporary guidance for the development and evaluation of complex interventions [45]. Standardised assessment of home practice and intervention fidelity will also be important to characterise actual intervention exposure. An appropriate comparator condition, longer-term follow-up, and prospectively defined progression criteria would allow future studies to distinguish intervention-specific effects from natural recovery and non-specific influences, and to determine whether the encouraging longitudinal symptom changes observed in this pilot translate into clinically meaningful benefits.

### 4.1. Clinical Implications

The principal clinical implication of this study is that a structured, low-intensity Qigong programme could be delivered within an existing hospital-based integrative oncology service with high retention, favourable supervised-session adherence, no reported intervention-related adverse events, and positive participant acceptability. These findings support the practical viability of further evaluating Qigong within post-surgical breast cancer survivorship care.

The observed reductions in symptom burden are encouraging but remain exploratory. Accordingly, the present findings do not establish Qigong as an effective treatment for pain, fatigue, anxiety, vasomotor symptoms, or other specific complaints and should not be used to replace established symptom-management strategies. Rather, they provide preliminary information about potentially relevant outcomes and demonstrate that a controlled evaluation of the programme appears practicable.

From an implementation perspective, the use of a standardised instructor-led programme, adaptation to individual functional limitations, scheduling compatible with work and family commitments, and the availability of home-practice materials represent potentially useful elements for future study design. A subsequent trial should determine which of these features are necessary to maintain adherence, as well as whether the programme remains feasible when delivered across different centres and instructors.

### 4.2. Study Limitations

Despite the encouraging findings, several limitations must be acknowledged that might temper their interpretation. Notably, Qigong encompasses a wide range of techniques and styles, and no universally standardised protocol, or clearly defined approach currently exists within mind–body intervention research. The absence of a control group limits the ability to attribute observed changes to Qigong, as spontaneous post-surgical recovery, concurrent oncological treatment, expectation effects, social interaction, and other non-specific influences may have contributed. The small sample size (n = 14) limits subgroup analyses, and the short-term follow-up leaves open questions about the long-term durability. In addition, no adjustment for multiple comparisons was applied to the secondary, symptom-specific or subgroup analyses. Therefore, the *p*-values obtained from these exploratory analyses should be interpreted with caution as they are only hypothesis-generating, given the increased risk of type I error. Furthermore, individual characteristics, including participant motivation, physical capacity, expectations regarding the practice, and instructor-related factors, may also influence engagement with the intervention and perceived outcomes of Qigong. Reliance on self-reported measures for home practice adherence may introduce recall or social desirability bias. However, these methodological choices are consistent with the exploratory aims of a feasibility-focused pilot study conducted in a real-world clinical setting.

An additional limitation is that symptom assessment was performed using a study-specific questionnaire adapted from the ESAS and SF-12 frameworks, rather than the original questionnaires. Although this approach allowed the inclusion of clinically relevant symptoms frequently encountered in routine survivorship care, the adapted questionnaire has not undergone formal psychometric validation. Therefore, the observed findings should be interpreted as exploratory and require confirmation using fully validated outcome measures in future controlled studies. Finally, this pilot feasibility study was not prospectively registered in a public clinical trial registry, which represents an additional reporting limitation.

Nevertheless, these limitations are common in the existing literature, where many studies have small sample sizes, short follow-up periods, and lack methodological rigour. Importantly, the safety profile of Qigong remains excellent, with no serious adverse events reported across studies. Moreover, women in different stages of cancer treatment and recovery, including older adults and those with comorbidities, can benefit from the programme’s low-intensity and flexible approach. In conclusion, further research into Qigong as a component of integrative oncology care is both justified and needed given its high acceptability and potential to improve several aspects of survivorship QoL.

## 5. Conclusions

This single-arm pilot study supports the feasibility, acceptability, and short-term safety of delivering an 8-week Qigong programme within a hospital-based integrative oncology service for women in the post-surgical phase of breast cancer care. Complete retention, high supervised-session adherence, absence of reported intervention-related adverse events, and favourable participant experiences indicate that the intervention and study procedures were generally practicable in this setting.

Overall patient-reported symptom burden also decreased during the study period; however, these exploratory pre–post changes cannot be attributed specifically to Qigong in the absence of a concurrent comparator.

These findings provide preliminary data to inform the design of larger, controlled studies and suggest that symptom burden, particularly across emotional and physical domains, represents a relevant outcome for future investigations of Qigong in breast cancer survivorship. Adequately powered randomised controlled trials with appropriate comparator groups, longer follow-up, validated patient-reported outcomes, objective functional measures, and, where relevant, biological or stress-related biomarkers are needed to determine the magnitude, clinical relevance, and durability of any Qigong-specific effects, as well as to investigate potential predictors and mechanisms of response.

## Figures and Tables

**Table 1:** Clinical and demographic data of BC Patients cohort.

Patient	Age	BMI (Kg/m^2^)	Body Fat (%)	Histology Tumour	Grade	Hormonal Status	Surgery	Menopause	Therapy	Smoking	Alcohol
P1	46	25.48	35.10	Ductal	III	HR^+^	Mastectomy	No	Tamoxifen	No	No
P2	73	27.43	43.10	Lobular	II	HR^+^	Mastectomy	Yes	Letrozole	No	Yes
P3	66	27.20	28.30	Ductal	II	HR^+^	Quadrantectomy	Yes	Letrozole	No	No
P4	59	24.49	29.80	NST Ductal	II	HR^+^	Quadrantectomy	Yes	Letrozole	No	No
P5	58	27.55	17.00	NST Ductal	II	HR^+^	Quadrantectomy	Yes	Letrozole, Abemaciclib	No	No
P6	42	18.24	15.40	Ductal	III	HR^+^	Quadrantectomy	No	Tamoxifen, Abemaciclib	No	No
P7	48	25.04	18.70	NST Ductal	II	HER2^+^	Quadrantectomy	Yes	Pertuzumab, Trastuzumab, Docetaxel	No	No
P8	49	20.13	23.90	Ductal	III	HR^+^, HER2^+^	Mastectomy	Yes	Pertuzumab, Trastuzumab, Docetaxel	No	No
P9	62	34.24	40.40	NST Ductal	II	HR^+^	Quadrantectomy	Yes	Letrozole	No	No
P10	66	23.78	24.00	Ductal	III	HR^+^	Quadrantectomy	Yes	Tamoxifen	No	Yes
P11	65	20.31	20.10	NST Ductal	II	HR^+^, HER2^+^	Quadrantectomy	Yes	Pertuzumab, Trastuzumab, Docetaxel	No	No
P12	48	22.59	30.90	NST Ductal	II	TNBC	Quadrantectomy	Yes	Pembrolizumab	No	Yes
P13	62	23.44	25.10	Ductal	III	HR^+^	Mastectomy	Yes	Letrozole	No	No
P14	54	23.54	20.90	Ductal	II	HR^+^	Mastectomy	Yes	Letrozole	No	No

Abbreviations: BMI, body mass index; NST, No Special Type; HR, hormone receptor; HER2, human epidermal growth factor receptor 2; TNBC, triple negative breast cancer.

**Table 2:** Individual BC patient-reported symptoms at baseline (T1).

Patient	Age	Patient-Reported Symptoms					Physical Activity		Emotional State				
		Changes in Appetite	Dysphagia/Nausea	Change in Bowel Habits	Hot Flushes	Pain	Tachycardia	Physical Activity	Fatigue	Mood Changes	Sleep Disturbances	Anxiety	Melancholy
P1	46	0	0	0	5	0	0	Walking	5	0	0	5	0
P2	73	1	1	1	0	3	5	None	0	5	4	4	5
P3	66	2	3	2	2	3	0	None	3	4	5	4	4
P4	59	3	4	3	3	5	0	None	4	4	3	3	4
P5	58	0	2	4	1	4	5	None	4	3	2	5	2
P6	42	1	0	4	4	5	0	None	1	5	3	1	0
P7	48	4	4	5	5	3	0	None	3	2	5	3	1
P8	49	0	1	0	4	1	0	None	2	3	0	5	3
P9	62	5	1	3	3	5	5	Walking	5	4	1	4	4
P10	66	1	0	0	4	0	0	Walking	2	0	2	2	0
P11	65	0	2	2	0	1	0	None	5	1	1	3	2
P12	48	5	5	5	5	5	0	None	0	5	0	5	0
P13	62	1	2	0	2	2	0	None	1	4	1	4	2
P14	54	2	5	5	3	4	0	None	4	2	0	3	0

**Table 3:** Individual BC patient-reported symptoms after the 8-week Qigong intervention (T2).

Patient	Age	Patient-Reported Symptoms					Physical Activity		Emotional State				
		Changes in Appetite	Dysphagia/Nausea	Change in Bowel Habits	Hot Flushes	Pain	Tachycardia	Physical Activity	Fatigue	Mood Changes	Sleep Disturbances	Anxiety	Melancholy
P1	46	0	0	0	2	0	0	Walking	2	0	0	2	0
P2	73	1	1	1	0	3	5	None	0	2	2	1	2
P3	66	0	2	1	1	1	0	None	3	1	4	0	3
P4	59	3	3	3	3	2	0	None	2	3	1	3	4
P5	58	0	1	4	1	4	5	None	4	3	1	3	1
P6	42	1	0	1	4	2	0	None	0	3	2	1	0
P7	48	4	3	2	4	3	0	None	3	1	5	3	0
P8	49	0	1	0	3	1	0	None	0	3	0	4	3
P9	62	2	0	3	1	4	5	Walking	2	2	1	2	2
P10	66	1	0	0	2	0	0	Walking	1	0	1	0	0
P11	65	0	1	2	0	0	0	None	4	1	0	3	0
P12	48	3	4	5	5	5	0	None	0	4	0	5	0
P13	62	1	1	0	1	1	0	None	1	2	1	2	1
P14	54	1	5	4	3	2	0	None	2	1	0	1	0

**Table 4:** Exploratory changes in symptom severity and prevalence between baseline (T1) and the end of the 8-week intervention (T2).

Outcome	T1 Median [IQR]	T2 Median [IQR]	HL Paired Shift (95% CI)	Wilcoxon (W)	Effect Size, r	T1 ≥ 3, n (%)	T2 ≥ 3, n (%)	Δ Prevalence (pp)	Exploratory Paired Test
Overall symptom burden	28.5 [19.0–34.25]	17.0 [11.75–26.25]	−8.0 [−10.5, −6.5]	0	0.88	—	—	—	Wilcoxon W = 0; *p* = 0.00012; r = 0.88
Appetite changes	1.0 [0.25–2.75]	1.0 [0–1.75]	0.0 [−1.0, 0.0]	0	0.49	4 (28.6)	3 (21.4)	−7.1	McNemar *p* = 1.000
Dysphagia/nausea	2.0 [1.0–3.75]	1.0 [0.25–2.75]	−0.5 [−1.0, 0.0]	0	0.76	5 (35.7)	4 (28.6)	−7.1	McNemar *p* = 1.000
Bowel-habit changes	2.5 [0.25–4.0]	1.5 [0.25–3.0]	0.0 [−1.5, 0.0]	0	0.50	7 (50.0)	5 (35.7)	−14.3	McNemar *p* = 0.500
Hot flushes	3.0 [2.0–4.0]	2.0 [1.0–3.0]	−0.5 [−1.5, 0.0]	0	0.65	9 (64.3)	6 (42.9)	−21.4	McNemar *p* = 0.250
Pain	3.0 [1.25–4.75]	2.0 [1.0–3.0]	−1.0 [−1.5, 0.0]	0	0.64	9 (64.3)	5 (35.7)	−28.6	McNemar *p* = 0.125
Tachycardia	0.0 [0–0]	0.0 [0–0]	0.0 [0, 0]	—	—	3 (21.4)	3 (21.4)	0.0	McNemar *p* = 1.000
Fatigue	3.0 [1.25–4.0]	2.0 [0.25–2.75]	−1.0 [−1.5, 0.0]	0	0.68	8 (57.1)	4 (28.6)	−28.6	McNemar *p* = 0.125
Mood changes	3.5 [2.0–4.0]	2.0 [1.0–3.0]	−1.0 [−1.5, −0.5]	0	0.72	9 (64.3)	5 (35.7)	−28.6	McNemar *p* = 0.125
Sleep disturbances	1.5 [0.25–3.0]	1.0 [0–1.75]	−0.5 [−1.0, 0.0]	0	0.66	5 (35.7)	2 (14.3)	−21.4	McNemar *p* = 0.250
Anxiety	4.0 [3.0–4.75]	2.0 [1.0–3.0]	−1.5 [−2.0, −1.0]	0	0.73	12 (85.7)	6 (42.9)	−42.9	McNemar *p* = 0.031
Melancholy	2.0 [0–3.75]	0.5 [0–2.0]	−0.5 [−1.5, 0.0]	0	0.65	5 (35.7)	3 (21.4)	−14.3	McNemar *p* = 0.500

Abbreviations: pp, percentage points; IQR, interquartile range; HL, Hodges–Lehmann.

## Data Availability

The original contributions of this study are included in the article. Further inquiries can be directed at the corresponding author.
